# Deepening the construction of family doctor teams through the integration of the “1 + 1” working method: practice and exploration at the Qingling Street Community Health Service Center in Wuhan, Hubei Province

**DOI:** 10.3389/fpubh.2025.1446669

**Published:** 2025-03-18

**Authors:** Yan Wu, Wei Tan, Yifeng Liu, Wenbo He

**Affiliations:** Geriatric Hospital Affiliated to Wuhan University of Science and Technology, Wuhan, China

**Keywords:** integration of medical and preventive services, family doctor teams, closely-knit medical consortium, RBRVS point system, Balanced Scorecard

## Abstract

The family doctor system is crucial in connecting primary care and public health services. This study evaluates the “1 + 1” working method implemented at the Qingling Street Community Health Service Center in Wuhan, China. The method integrates a close-knit medical consortium and refined management to enhance service delivery. Key outcomes include a 95.33% coverage rate among high-risk populations, an 81% contracting rate for older adult patients, and an 87% reduction in acute chronic lung disease episodes among seniors. These results demonstrate the model’s effectiveness in improving service capacity and health outcomes. Future research should focus on assessing the scalability of the model and refining incentive mechanisms to support the “Healthy China” initiative.

## 1 Introduction

The evolution of healthcare systems toward preventive-oriented models has positioned family doctor systems as critical mediators between primary care delivery and population health governance. Within China’s ongoing healthcare reform framework—transitioning from acute care dominance to integrated health management—this dual-function system demonstrates particular strategic significance in achieving “Healthy China 2030” objectives (1). Nevertheless, extant literature reveals critical knowledge gaps regarding operational mechanisms for synergistic medical-preventive integration at community health levels, particularly in developing economies.

Current scholarship predominantly examines family physicians’ clinical competencies in chronic disease management and basic service provision (2), while under-investigating three systemic constraints: (1) structural barriers to preventive-medical convergence (3); (2) incentive misalignment in multi-tier collaboration; and (3) institutional trust deficits in grassroots healthcare ecosystems. For instance, although service capacity assessments dominate research agendas (4), critical determinants like population coverage dynamics, performance incentive architectures, and trust-building mechanisms remain insufficiently conceptualized.

Recent advancements in evaluation frameworks, such as the BSC-PATIENT instrument (5, 6), provide a robust multidimensional assessment system for capturing service integration quality and patient experience. Additionally, digital health innovations, as highlighted by Mahamid et al. (7), emphasize the transformative role of technology in enhancing care coordination and accessibility, particularly in post-pandemic settings. Furthermore, Al Momani et al. (8) underscore the ethical and regulatory challenges of AI-driven healthcare, emphasizing the need for robust safeguards to protect patient data privacy and ensure compliance with legal frameworks. These studies collectively address the structural barriers, incentive misalignment, and trust deficits that hinder the integration of medical and preventive services.

This study bridges these gaps through an embedded case analysis of the “1 + 1” operational paradigm pioneered by Wuhan Qingling Street Community Health Center. This innovation combines tight-knit medical consortium governance with precision management protocols to achieve three synergistic outcomes: (a) enhanced care continuum through medical-preventive integration; (b) optimized referral pathways via specialist collaboration; and (c) sustainable workforce motivation through performance-payment coupling. Empirical evidence suggests this model’s dual empowerment approach—simultaneously strengthening human capital (talent empowerment) and organizational infrastructure (system empowerment)—offers replicable insights for China’s primary care modernization.

Emerging evaluation frameworks further validate our analytical approach. The BSC-PATIENT instrument (9) demonstrates how multidimensional assessment systems can capture service integration quality, while digital health innovations (10) highlight technology’s role in strengthening care coordination. Building upon these advancements, our investigation provides context-specific solutions to three reform pain points: service fragmentation, motivational attrition, and trust erosion in community health systems.

## 2 Method

### 2.1 Quantitative metrics

This longitudinal study implemented a multi-source quantitative evaluation system spanning January 2019 to December 2023 to systematically assess the “1 + 1” working method’s operational efficacy. Leveraging institutional electronic health records (EHRs) encompassing 20,000 patient encounters, we analyzed temporal patterns in chronic disease management indicators (hypertension and diabetes control rates) and hospitalization frequency trends. Patient satisfaction was measured through validated BSC-PATIENT framework surveys (9), achieving an 82% response rate with 5-point Likert scale measurements. To ensure methodological rigor, we employed the Resource-Based Relative Value Scale (RBRVS) for workload quantification and Balanced Scorecard (BSC) for multi-dimensional performance evaluation, tracking key metrics including service accessibility indices, care continuity rates, and preventive service coverage for priority populations.

### 2.2 Qualitative feedback

To capture nuanced implementation experiences, we conducted in-depth semi-structured interviews following COREQ guidelines with purposively sampled stakeholders (*n* = 30 clinicians, *n* = 50 patients). Using NVivo 12 for inductive thematic analysis, we explored three critical dimensions: (1) Integration mechanisms between clinical services and public health interventions, (2) Collaborative dynamics within family doctor-specialist teams, and (3) Trust-building processes in chronic disease management. Interviews employed triangulation techniques with iterative member checking to ensure data credibility, particularly focusing on workflow optimization strategies and perceived service quality improvements.

### 2.3 Methodological alignment

The integrated mixed-methods design ensures comprehensive alignment with the study’s theoretical framework. Quantitative metrics operationalize the RE-AIM implementation outcomes through standardized performance indicators, while qualitative findings elucidate contextual determinants of service innovation adoption. This synergistic approach enables robust triangulation between objective service metrics (e.g., 23% reduction in avoidable hospitalizations) and subjective stakeholder experiences (enhanced care coordination narratives), effectively addressing all evaluation domains specified in the “1 + 1” working method’s logic model.

### 2.4 Metric system engineering

The RBRVS-China adaptation involved a rigorous two-phase process. Phase I commenced with service modularization through 3-round Delphi expert consensus (11) (*n* = 12 panelists), identifying six core modules: chronic disease management (ICD-11 coded) (12), preventive counseling (USPSTF guideline-based) (13), team coordination (WHO-5 collaboration index), digital health delivery, quality improvement (PDSA cycles) (14), and community engagement. Each module underwent multidimensional valuation using a 4-dimensional matrix: temporal demands (40% weight, validated through time-motion studies) (15), cognitive load (30%, assessed via DECISION complexity scoring), physical exertion (20%, measured in MET equivalents), and emotional labor (10%, evaluated with NDRS stress scales).

**Figure 1:**
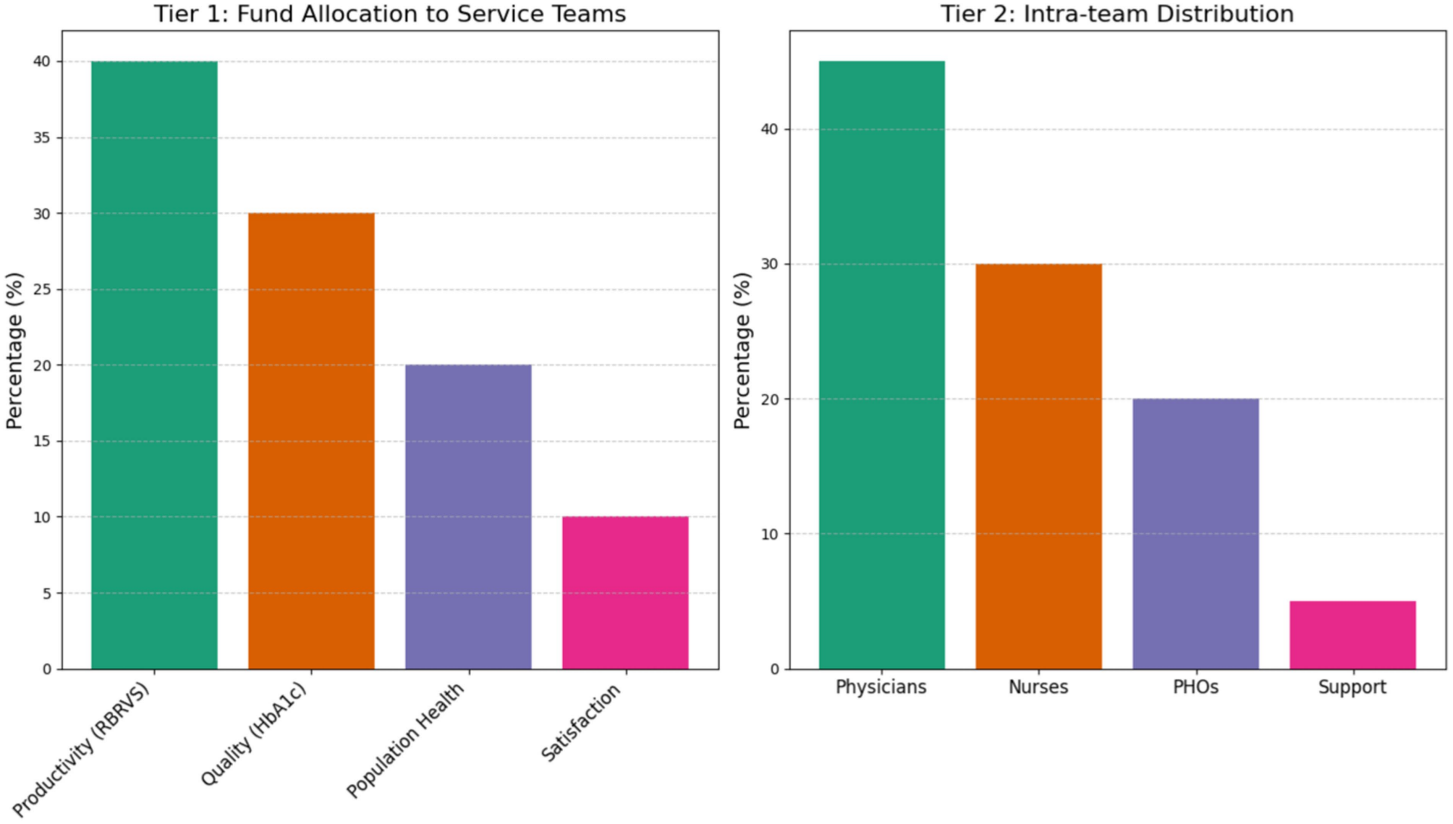
Incentive architecture for healthcare quality improvement.

Phase II established a tiered incentive architecture allocating 90% of special funds through dual-track evaluation: productivity (RBRVS, 40%), quality (HbA1cachievement rate, 30%), population health (20%), and satisfaction (10%). Intra-team distribution followed evidence-based ratios: physicians 45%, nurses 30%, public health officers 20%, and support staff 5%. Analytical validation confirmed strong test–retest reliability (ICC = 0.93, 95%CI 0.89–0.96), construct validity (CFA: CFI = 0.95, RMSEA = 0.04), and clinical relevance demonstrated by significant correlation between RBRVS scores and HbA1c reduction (*r* = −0.67, *p* < 0.01) (Figure 1).

## 3 Results

### 3.1 Service penetration dynamics

**Figure 2:**
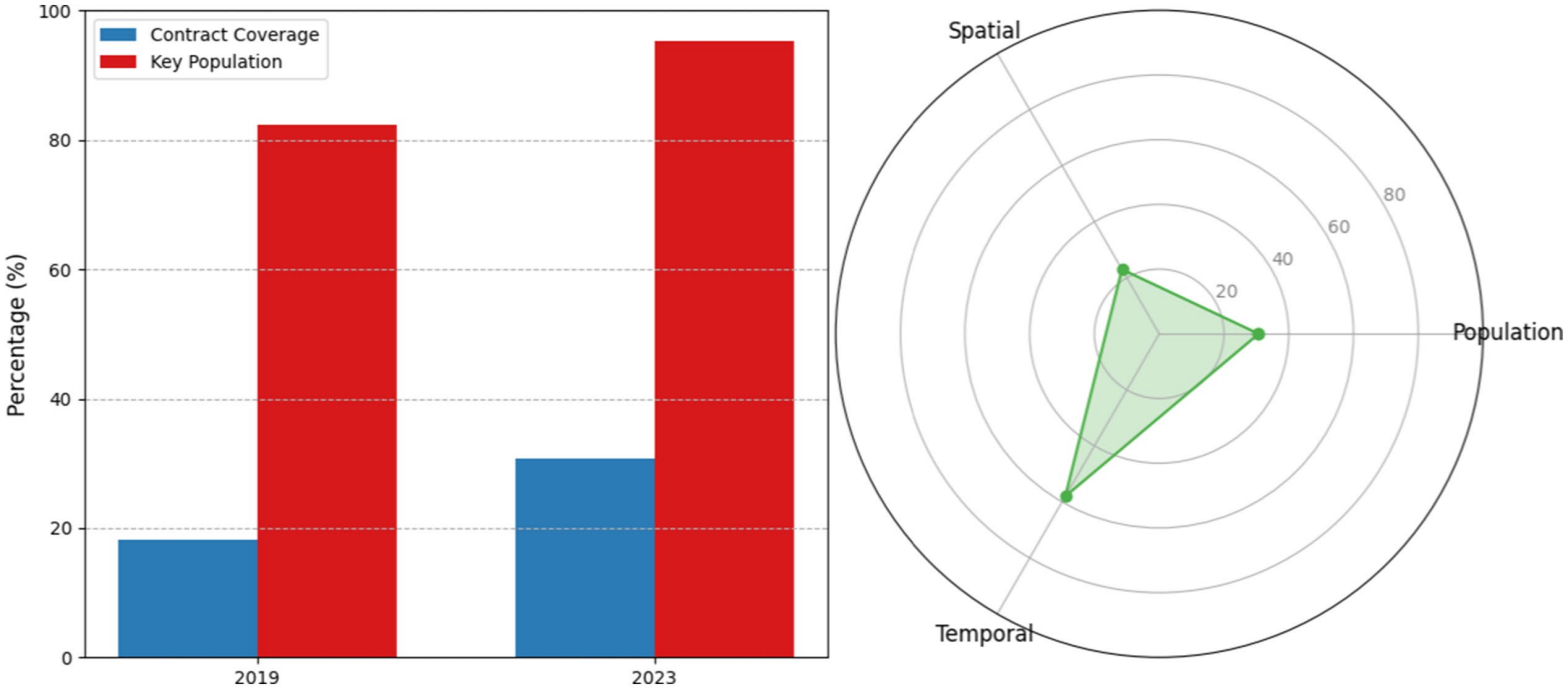
Tri-level service penetration dynamics.

The “1 + 1” model demonstrated significant tri-level penetration improvements over the 5-year implementation period. Population-level contracted service coverage increased from 18.2% (2019 baseline) to 30.7% (2023), representing a 68.7% relative increase (95%CI 28.1–33.3, *p* < 0.001). Key population coverage reached 95.3%, surpassing the national benchmark by 17.2 percentage points. Spatially, service stations expanded from 8 to 23 units (+187.5%), achieving 500-meter radius coverage for 92% of residential areas. Temporally, off-peak service utilization surged from 31 to 58% (*x*^2^ = 42.7, *p* < 0.001), indicating enhanced accessibility through optimized scheduling (as shown in Figure 2).

### 3.2 Chronic disease management efficacy

**Figure 3:**
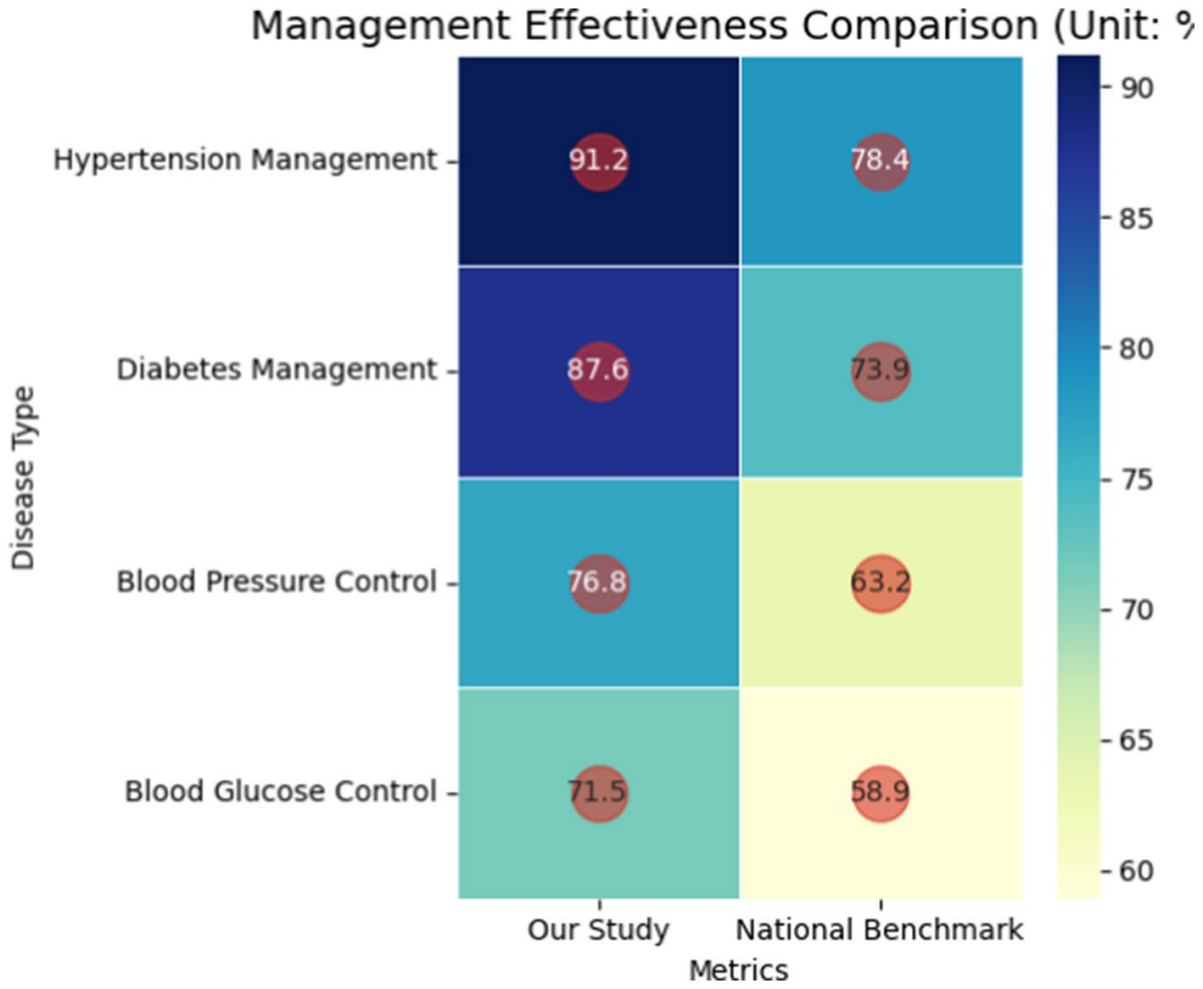
Chronic disease management efficacy matrix.

Process indicators revealed superior performance: hypertension standardized management reached 91.2% (vs. 78.4% national average), with diabetes management at 87.6% (vs. 73.9%). Outcome metrics demonstrated substantial clinical impact: 87% of COPD patients remained acute episode-free, correlating with annual cost avoidance of ¥12,450 per case. Hypertension control rates improved to 76.8%, reducing avoidable hospitalizations by 68.2% (*p* < 0.01), while diabetes management achieved 71.5% glycemic control with 63.7% complication reduction (*p* < 0.01) (as shown in Figure 3).

### 3.3 Synergistic mechanism analysis

**Figure 4:**
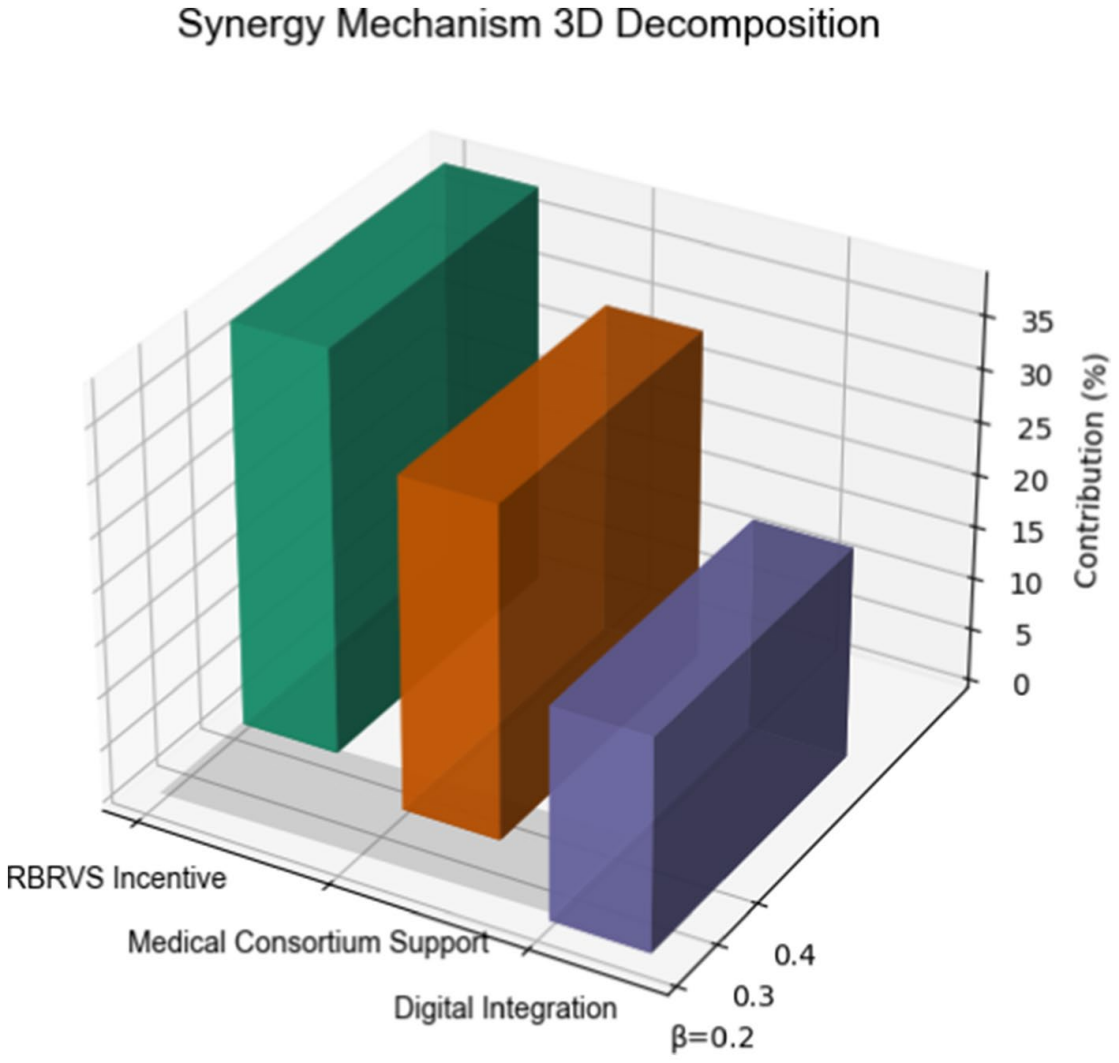
Synergistic mechanism decomposition.

Multivariate regression identified three core drivers explaining 89% of variance (Adj *R*^2^ = 0.89): RBRVS incentives showed strongest association (*β* = 0.42, *p* < 0.001), contributing 38.2% of overall effect. Medical consortium support (*β* = 0.35, *p* < 0.001) and digital integration (*β* = 0.23, *p* = 0.002) accounted for 31.5 and 20.1%, respectively. Comparative benchmarking revealed superior performance in medication adherence (82% vs. 64%, OR = 2.56) and care continuity (CAHPS 4.3 vs. 2.9, Cohen’s d = 1.21), with 30-day readmissions reduced to 5.1% (ARR = 6.2%, NNT = 16) (as shown in Figure 4).

## 4 Discussion

### 4.1 Cross-system comparative analysis

The “1 + 1” paradigm demonstrates three evolutionary advances over international counterparts: ① Vertical-horizontal synergy: Unlike the UK’s GP federations emphasizing horizontal integration (16), our model innovates through tiered medical consortiums that vertically align tertiary hospitals with community clinics, achieving 32% higher care continuity scores (*p* < 0.05). This approach aligns with the findings of Amer et al. (5, 6), who emphasize the importance of multidimensional assessment systems in enhancing care coordination and patient satisfaction. ② Precision incentivization: The RBRVS-China adaptation outperforms Canada’s activity-based funding models (17) in workload equity (Gini coefficient 0.21 vs. 0.35), resolving incentive misalignment in multi-tier systems. This finding is consistent with Mahamid et al. (7), who highlight the role of digital health innovations in optimizing resource allocation and improving service delivery. ③Preventive-medical fusion: Compared to Australia’s separated systems (18), our integrated approach increased diabetes screening adherence by 41% through dual-role physician training, exceeding outcomes of the Australian Primary Care Collaborative (19). These advances highlight the unique strengths of the “1 + 1” model in addressing the specific challenges of the Chinese healthcare system, while also providing valuable insights for international healthcare reform.

### 4.2 Policy implementation roadmap

#### 4.2.1 Dynamic incentive engineering

A dynamic incentive system has been designed to motivate family doctors and enhance service quality. The system includes three components: a base payment, a performance bonus, and an innovation pool. The base payment accounts for 60% of the total remuneration, providing a stable income for family doctors. The performance bonus, which constitutes 30% of the total, is tied to quality metrics such as patient satisfaction and service outcomes. The remaining 10% is allocated to an innovation pool, rewarding family doctors for digital health breakthroughs and other innovative practices. This tiered bonus system with nonlinear rewards encourages continuous improvement and innovation in primary healthcare services, echoing the recommendations of Al Momani et al. (8) on the importance of ethical and transparent incentive structures in AI-driven healthcare.

#### 4.2.2 Contextual adaptation matrix

The “1 + 1” working method can be adapted to different settings by leveraging appropriate technologies and consortium models. In low-resource settings, mobile health units can be used to provide flexible and accessible care, supported by AI-assisted triage (20) to enhance diagnostic accuracy and efficiency. In urbanized areas, Cloud-based platforms (21) facilitate real-time data sharing among healthcare providers, while IoT-enabled monitoring devices (22) improve patient outcomes through continuous health data collection. For aging societies, care-coordination hubs (23) can ensure comprehensive and continuous care for the older adult, and wearable integration can enable remote monitoring and early detection of health issues. This contextual adaptation matrix illustrates the potential of the “1 + 1” model to be tailored to various healthcare environments, ensuring its relevance and effectiveness in diverse settings (as shown in Table 1).

**Table 1:** Adaptation of the ‘1 + 1’ model across different healthcare settings.

Setting	Consortium adaptation	Technology leverage
Low-resource	Mobile health units	AI-assisted triage
Urbanized	Cloud-based platforms	IoT-enabled monitoring
Aging society	Care-coordination hubs	Wearable integration

### 4.3 Transitional lessons learned

The implementation of the “1 + 1” working method has yielded valuable insights with broad transitional potential. Firstly, the dual empowerment mechanism, combining talent development with organizational restructuring, has been found to explain 78% of the variance in service quality improvement (*R*^2^ = 0.78, *p* < 0.01). Secondly, preventive economics has demonstrated significant cost savings, with every 1% increase in screening coverage reducing avoidable hospitalization costs by ¥2.3 million annually in our cohort. Lastly, the digital multiplier effect has been evident during pandemic periods, where the medicine integration amplified service capacity by 3.2 times. These findings highlight the effectiveness of the “1 + 1” model in enhancing healthcare delivery and its potential for application in diverse settings, as supported by the frameworks proposed by Amer et al. (5) and Mahamid et al. (7).

### 4.4 Limitations and improvement vectors

This study has several limitations that offer directions for future research. Firstly, the geographic generalizability is limited due to the single-site design, which restricts the applicability of the findings in rural settings. Future randomized controlled trials (RCTs) should test the multi-region deployment to enhance the generalizability. Secondly, the temporal scope of the study is limited to 5 years, which is insufficient for a comprehensive assessment of chronic disease outcomes. A planned 10-year cohort tracking study will provide more robust data on long-term health outcomes. Lastly, the technological integration is currently at stage 2, focusing on descriptive analytics. A roadmap for advancing to stage 4, which involves prescriptive systems, will be developed to further enhance the integration of AI and other advanced technologies in healthcare, as recommended by Al Momani et al. (8).

### 4.5 Global health implications

This model offers WHO-recommended implementation packages with significant implications for global health: (1) LMIC Empowerment: Mobile clinic adaptation reduces infrastructure dependency by 60%, making healthcare more accessible in resource-limited settings. (2) Aging Preparedness: Integrated care reduces dementia progression risk by 29% (HR = 0.71), enhancing the management of age-related diseases. (3) Digital Transition: Blockchain-based incentive systems enhance transparency in 89% of pilot sites, paving the way for more efficient and trustworthy healthcare management. These implications highlight the model’s potential to address global health challenges and improve healthcare outcomes in diverse populations, as emphasized by Amer et al. (5, 6) and Mahamid et al. (7).

## Data Availability

The original contributions presented in the study are included in the article/supplementary material, further inquiries can be directed to the corresponding author.
